# 161. Flucytosine Utilization and Dosing Practices at an Academic Medical Center

**DOI:** 10.1093/ofid/ofab466.363

**Published:** 2021-12-04

**Authors:** Molly M Miller, Emily Kreikemeier, Erica J Stohs, Trevor C Van Schooneveld, Trevor C Van Schooneveld, Bryan Alexander, Scott J Bergman, Scott J Bergman

**Affiliations:** 1 Nebraska Medicine, Bellevue, Nebraska; 2 University of Nebraska Medical Center, Omaha, NE

## Abstract

**Background:**

The typical dose of flucytosine is 25 mg/kg/dose every 6 hours for severe infections due to *Candida* and *Cryptococcus*. Many hospital protocols use ideal body weight (IBW) for initial dosing to achieve a goal peak serum concentration of 30-80 mcg/mL, but this is supported by very limited data. Our objective was to evaluate flucytosine dosing strategies, describe safety concerns, and explore financial benefits associated with using IBW.

**Methods:**

All inpatient flucytosine orders for adults from 1/1/2015 through 10/31/2020 were retrospectively evaluated. Doses, weight used, flucytosine levels, adverse events, and potential cost savings associated with IBW dosing were characterized.

**Results:**

During this period, 35 patients received flucytosine. The most common indications were cryptococcal meningitis (73%), pulmonary cryptococcosis (14%), and candidiasis (11%). Most patients were receiving concurrent liposomal amphotericin B (92%). Based on body mass index, most patients were overweight or obese (60%). Actual body weight was used for initial dosing in most cases (81%). Flucytosine peak monitoring was performed in 51% of cases. Initial peak levels were supratherapeutic in 10/19 cases (53%). Of those 10 patients, 70% were overweight/obese, and 60% would have received a lower initial dose if IBW had been used with dose rounding to the nearest 500mg capsule. Adverse events for all patients included new onset cytopenias, hepatic and renal dysfunction, occurring in 20%, 11%, and 60% respectively. Those with supratherapeutic levels had higher rates of new onset hepatic and renal dysfunction, 30% and 90% respectively. In 32% of cases, using IBW would have resulted in a lower daily dose, with an average dose reduction of 1888 mg, resulting in a mean cost savings per patient of &640/day using average wholesale price.

**Conclusion:**

Most flucytosine orders were not dosed using IBW, which may have led to supratherapeutic levels. Using IBW for dosing in overweight patients may lead to reduced toxicity and potential cost savings. The default dosing weight for flucytosine in our electronic medical record will be set to IBW to encourage change.

**Disclosures:**

**Trevor C. Van Schooneveld, MD, FACP**, BioFire (Individual(s) Involved: Self): Consultant, Scientific Research Study Investigator; Insmed (Individual(s) Involved: Self): Scientific Research Study Investigator; Merck (Individual(s) Involved: Self): Scientific Research Study Investigator; Rebiotix (Individual(s) Involved: Self): Scientific Research Study Investigator **Bryan Alexander, PharmD**, **Astellas Pharma** (Advisor or Review Panel member) **Scott J. Bergman, PharmD, FCCP, FIDSA, BCPS, BCIDP**, **Merck & Co., Inc** (Grant/Research Support) **Scott J. Bergman, PharmD, FCCP, FIDSA, BCPS, BCIDP**, Merck & Co., Inc (Individual(s) Involved: Self): Research Grant or Support

